# A Triple Oral Combination of Bendamustine, Acalabrutinib, and Venetoclax Demonstrates Efficacy Against Mantle Cell Lymphoma In Vitro and In Vivo

**DOI:** 10.3390/cancers17111889

**Published:** 2025-06-05

**Authors:** Dimitrios Filioglou, Nina Santa-Cruz, Geovana S. F. Leite, Dan W. Davini, Megan J. Cracchiolo, Forrest L. Baker, Muhammad Husnain, Richard J. Simpson, Vasilios Voudouris, Emmanuel Katsanis

**Affiliations:** 1 Department of Pediatrics, University of Arizona, Tucson, AZ 85721, USA; 2 School of Nutritional Sciences and Wellness, University of Arizona, Tucson, AZ 85721, USA; 3 University of Arizona Cancer Center, University of Arizona, Tucson, AZ 85721, USA; 4 Department of Medicine, University of Arizona, Tucson, AZ 85721, USA; 5 Department of Immunobiology, University of Arizona, Tucson, AZ 85721, USA; 6 Exinda Therapeutics LLC, El Dorado Hills, CA 95762, USA

**Keywords:** oral bendamustine, mantle cell lymphoma, novel drug formulation, preclinical models

## Abstract

**Background/Objectives**: Bendamustine (BEN) combined with rituximab (RTX) remains a standard first-line therapy for transplant-ineligible patients with newly diagnosed mantle cell lymphoma (MCL). Meanwhile, novel targeted therapies such as Bruton tyrosine kinase inhibitors (BTKis) are increasingly used in the treatment of relapsed/refractory (R/R) MCL. We recently reported that a novel oral formulation of BEN exhibits comparable efficacy to the intravenous counterpart. In this study, we investigated the efficacy of oral BEN administered alone or in combination with the oral BCL-2 inhibitor Venetoclax (VEN) and/or the oral BTKi Acalabrutinib (ACAL), against two human MCL cell lines (Jeko-1 and Z-138) representative of the R/R disease subtype. **Methods**: We performed in vitro analyses using MTS viability and Annexin V/PI apoptosis assays. For the in vivo studies, all treatments were administered via oral gavage in xenograft mouse models. Therapeutic efficacy was evaluated by monitoring tumor growth and survival. **Results**: BEN induced significant cytotoxicity in both cell lines at low, clinically relevant concentrations. In contrast, VEN demonstrated limited efficacy as monotherapy, with Z-138 showing sensitivity only at high doses. However, combining BEN with VEN with or without ACAL, enhanced apoptosis and cytotoxicity, with more pronounced effects in Z-138. In vivo, oral BEN significantly reduced tumor growth and prolonged survival in both xenograft models. In the Z-138 model, the addition of VEN ± ACAL further improved survival outcomes. **Conclusions**: Our findings support the efficacy of oral BEN as both a monotherapy and as part of an all-oral treatment regimen for MCL. These results warrant further investigation into the clinical potential of oral BEN, particularly in combination with targeted agents.

## Simple Summary

Although oral targeted therapies have improved the treatment of mantle cell lymphoma (MCL), outcomes remain poor. We recently showed that an oral form of Bendamustine works as well as the intravenous version. In this study, we tested its effect—alone or in combination with two other oral drugs, Venetoclax and Acalabrutinib—on two human MCL cell lines. We found that oral Bendamustine strongly reduced tumor growth and improved survival in mice with human MCL, performing better than the other single treatments. When combined with Venetoclax, with or without Acalabrutinib, its anti-tumor effect was even stronger in a cell line-dependent manner. These findings support further investigations of oral Bendamustine as a treatment option in MCL.

## 1. Introduction

Mantle cell lymphoma (MCL) is an aggressive type of non-Hodgkin’s lymphoma (NHL) characterized by the malignant transformation of B lymphocytes found in the outer ring of the lymphatic follicle around the germinal center, known as the mantle zone. While the hallmark chromosomal translocation is the t (11;14) (q13; q32), resulting in cyclin D1 overexpression and consequent cell cycle dysregulation at the G1-S transition, MCL exhibits significant heterogeneity due to a variety of genetic and epigenetic alterations [1,2]. Prognosis remains generally poor despite the advances in chemo-immunotherapy (CIT) [3]. Bendamustine (BEN) is an alkylating agent with unique structural characteristics that contribute to its anticancer properties [4]. Intravenous (IV) BEN combined with anti-CD20 monoclonal antibody rituximab (RTX) is currently the first-line treatment for previously untreated patients because it prolongs progression-free survival, it is well-tolerated in the elderly population, and it offers a longer duration of response compared to other regimens such as the R-CHOP (rituximab, cyclophosphamide, doxorubicin, prednisone) and the R-CVP (rituximab, cyclophosphamide, vincristine, prednisone) [1,2,5,6]. A recent large Korean study reported that treatment with BEN + RTX resulted in a three-year overall survival (OS) of 92% in treatment-naïve patients and 66.8% in those with relapsed refractory (R/R) MCL [7]. Notably, BEN with or without RTX has shown both good tolerability and efficiency in R/R MCL, with the potential to overcome the frequent resistance observed after relapse [8].

Importantly, the overactivation of the B-cell receptor (BCR) signaling cascade has been found to play a critical role in the survival and the proliferation of neoplastic cells in MCL [9]. Inhibitors of Bruton tyrosine kinase (BTKi) have therefore become a cornerstone in the treatment of relapsed/refractory (R/R) MCL [10]. The first-generation BTKi ibrutinib (IBR) was introduced for the treatment of R/R MCL due to its high response rates [11]. However, studies have shown that IBR is frequently discontinued because of disease progression and common adverse events including bleeding, cytopenias and atrial fibrillation [12]. Acalabrutinib (ACAL) is a second-generation irreversible BTKi administered orally. Due to its higher selectivity for BTK, it has demonstrated a favorable safety profile, with lower rates of thrombocytopenia and atrial fibrillation compared to first-generation BTKi [13]. ACAL has been FDA-approved for the treatment of R/R MCL based on its ability to achieve high rates of durable responses and a favorable safety profile, both of which were maintained at a median 26-month follow-up [14,15]. Final efficacy results showed that ACAL achieved a median OS of 59.2 months and a median progression-free survival (PFS) of 22 months. Notably, the overall response rate (ORR) was high (80.8%), even among patients with blastoid/pleomorphic variants [16]. More recently, a study demonstrated that the incorporation of ACAL with Bendamustine and rituximab significantly improved PFS in transplant-ineligible patients with previously untreated MCL, without excess toxicity, thereby leading to the FDA approval of this combination for this group of patients [17].

The upregulation of the B-cell lymphoma 2 (BCL-2)-mediated antiapoptotic pathway has been associated with the progression of MCL. Venetoclax (VEN), an oral Bcl-2-specific inhibitor, induces TP-53 independent programmed cell death by disrupting the mitochondrial outer membrane [18]. Although MCL has demonstrated responses to monotherapy with VEN, the duration of the response has generally been limited [19]. Interestingly, studies have found that MCL cell lines resistant to IBR exhibit increased sensitivity to VEN due to BCL-2 upregulation [20]. Moreover, a recent study reported that combining IBR with VEN in patients with R/R MCL led to higher complete response rates (CRR) compared to either agent alone [21]. Wang et al. reported that combining ACAL, VEN and RTX was well-tolerated and achieved a 71.4% complete response (CR) rate along with high minimal residual disease (MRD) negativity in treatment-naïve MCL patients [22]. Similar promising results were observed with the combination of ACAL, VEN, and the newer monoclonal antibody Obinutuzumab [23]. In a recent phase I clinical trial, the combination of BEN, IBR, VEN and RTX was well-tolerated in patients with R/R MCL, achieving an ORR and CR of 80% [24].

Several combinations of targeted agents such as BTKis are currently being tested in clinical trials to determine whether chemotherapy-containing regimens can be reduced or even avoided. However, it remains unclear whether CIT or its combination with targeted therapies can achieve better outcomes compared to chemotherapy-free regimens [25]. Moreover, there is a growing trend in hematology/oncology toward shifting from traditional IV medications to oral formulations, which provide greater independence for patients [26]. Our group recently demonstrated that the administration of a novel oral form of BEN is as effective as IV administration in mediating anti-tumor effects [27]. In this study, we aimed to investigate the efficacy of this novel oral BEN either alone or in combination with other oral agents such as VEN and/or ACAL, which are currently being investigated in relapsed/refractory R/R MCL.

## 2. Materials and Methods

### 2.1. Cell Culture

Jeko-1 (CRL-3006) and Z-138 (CRL-3001) were obtained from ATCC (Manassas, VA, USA) and luciferase tagged Jeko-1 (Jeko-1-Luc) was purchased from FenicsBio (Halethorpe, MD, USA) (CL-1636). Cells were cultured in RPMI 1640 (SH30027.01, Cytiva, Marlborough, MA, USA) supplemented with 20% fetal bovine serum (FBS, Millipore, Burlington, MA) (or 10% for the Z-138), Penicillin-Streptomycin (Gibco, 15-140-148, Grand Island, NY, USA), HEPES (Gibco, 15630-080) and Sodium Pyruvate (Cytiva, SH30239.01) and maintained at 37 °C in a humidified incubator with 5% CO2. Cell density was monitored and adjusted every 1-2 days to maintain exponential growth. All cell lines were tested negative for Mycoplasma contamination by Lonza MycoAlert Mycoplasma Detection Kit (Lonza, Walkersville, MD, USA).

### 2.2. Reagents

For the purposes of in vitro experiments, ACAL (cat#: S8116) and BEN (cat#: S1212) were purchased from SelleckChem (Houston, TX, USA), while for the in vivo experiments BEN and ACAL were provided by Exinda Therapeutics (El Dorado Hills, CA, USA). Exinda sourced ACAL maleate monohydrate from Hetero Drugs Ltd. (Hyderabad, Telangana, India) . Oral BEN was obtained by the method described on example 4 of patent US11701344. It is a supersaturated amorphous solid dispersion (ASD) obtained through a manufacturing process based on spray drying. It is a dry powder that consists of micro-particles containing Bendamustine HCL monohydrate and polyvinylpyrrolidone PVP-K17 at a weight ratio of 1:3. VEN was purchased from either SelleckChem (cat#: S8048) or MedChem (Monmouth Junction, NJ, USA) (cat#: HY-15531). 

### 2.3. [3-(4,5-Dimethylthiazol-2-yl)-5-(3-carboxymethoxyphenyl)-2-(4-sulfophenyl)-2H-tetrazolium] (MTS) Assays

Here, 30,000 Jeko-1 or Z-138 were plated in a 96-well plate in the presence of media with or without BEN, VEN or ACAL. For the single-drug assays, each drug was diluted in media and then added in serial dilution in either duplicates or triplicates. For the combination assays with BEN, 1 micromolar (1 μM) of ACAL and/or VEN was added to the corresponding wells. Following a 72 h incubation at 37 °C and 5% CO_2_, plates were removed from the incubator and 20 μL of reagent was added to each well using the CellTiter 96 Aqueous One Solution Cell Proliferation Assay (VWR, Promega Corporation, Madison, WI, USA). Cells were further incubated at 37 °C and 5% CO_2_ and 4 h later plates were read using the BioTek Gen 5 Microplate Software at 490 nm (PowerWave XS, BioTek, Winooski, VT, USA). The normalized viability of all treated cells was calculated as a % of the untreated control cells. All experiments were repeated four independent times.

### 2.4. Annexin-PI Assays

Jeko-1 or Z-138 were seeded at a density of 30,000 cells per well in a 96-well plate and then treated with various BEN concentrations alone or in combination with 1 μM ACAL +/− 1 μM VEN. Then, 24 h later, the cells were harvested, washed, and then assessed with AlexaFluor-488 Annexin V/PI staining (Invitrogen, Carlsbad, CA, USA) according to the manufacturer’s instructions. The summary percentage (%) of apoptotic and dead cells was calculated by deducting the % of live cells (Annexin-V negative and PI-negative cells) from the total (100%). All experiments were repeated five independent times.

### 2.5. In Vivo Tumor Models

NSG (NOD.Cg-Prkdcscid Il2rgtm1Wjl/SzJ) mice were purchased from the Jackson Laboratory and bred at the University of Arizona Experimental Mouse Shared Resource. Female or male mice aged 6–13 weeks were distributed by equal average weight across treatments and used for experiments. For the intravenous model, following three washes in PBS, 10^4^ Jeko-1-Luc cells were intravenously injected in mice 24 h after a 150 cGy total body irradiation with a RadSource X-ray irradiator. For the subcutaneous model, 4 × 10^6^ Z-138 were resuspended in PBS and injected subcutaneously in the right flank. Oral (PO) BEN was weighed fresh for each experiment, first dissolved in DMSO, and then diluted with filtered water (H_2_O) to the final concentration of 30 mg/kg, and given orally on day +1 and day +8 following tumor inoculation. Venetoclax was weighed fresh for each experiment and dissolved directly into a vehicle composed of 60% phosal50-PG, 30% PEG-400 and 10% ethanol, and was then administered via oral gavage at 100 mg/kg on days +2, +5, +9, +12, +15, +18, +21, +24 and +27. Acalabrutinib maleate monohydrate was dissolved in 100% DMSO, stored at −80 °C, and diluted fresh on the day of dosing into a vehicle consisting of 2% DMSO, 30% PEG300, 2% Tween-80, and PBS. This solution was administered via oral gavage at 50 mg/kg on days +3, +4, +6, +7, +10, +11, +13, +14, +16, +17, +19, +20, +22, +23, +25, +26, +28 and +29. Each mouse was given the appropriate medication or the corresponding vehicle. Mice were weighed twice weekly and monitored daily for survival. Animals losing more than thirty percent of their starting weight for two consecutive measurements or experiencing total hind-limb paralysis were sacrificed. For the subcutaneous model, tumor measurement with calipers was repeated every 3–4 days. Tumor size was determined by taking a length (L) and width (W) measurement and calculating volume using the following equation: L × W^2^ × (π/6). Animals were euthanized when tumor volume exceeded 5000 mm^3^ or became ulcerated.

### 2.6. Bioluminescence Imaging

Bioluminescence imaging (BLI) was performed weekly to assess the tumor burden. Briefly, after receiving a 200 μL intraperitoneal (IP) injection of D-luciferin (15 mg/mL, GoldBio, St Louis, MO, USA), mice were anesthetized with 2% isoflurane, and then imaged using the Spectral Instruments Lago-X (Tucson, AZ, USA) system. BLI was quantified using the Aura imaging software and presented as photons/s in a region of interest that includes the entire animal. BLI images were gathered following a 5-min exposure. For visual representation, scales were adjusted to a radiance scale minimum of 1.7 × 10^4^ and maximum of 5.5 × 10^7^ and saved as JPEG files.

### 2.7. Statistical Analysis

All statistical analyses were performed with the use of GraphPad Prism V.10. One-way ANOVA with Tukey’s post-hoc analysis was used to assess the differences between culture conditions in the Annexin-PI cytotoxicity assays. For the MTS assays, half-max inhibitory concentrations (IC50) with 95% confidence intervals (95% CI) for each agent were calculated with the use of nonlinear fit [Inhibitor] vs. response—variable slope (four parameter). Kaplan–Meier survival curves derived with the Log-rank Mantel–Cox test were used for the survival analysis of the in vivo experiments. BLI data in the IV mouse model were compared with a mixed linear mode using Tukey’s post-hoc analysis. Unless stated otherwise, data represent the mean +/− standard error of the means (S.E.M.). *p* values of <0.05 were considered significant. The asterisks show increasing levels of significance (* <0.05, ** <0.01, *** <0.001, **** <0.0001).

## 3. Results

### 3.1. Sensitivity of MCL Cell Lines to Bendamustine, Venetoclax and Acalabrutinib Monotherapies and Combination Therapies In Vitro

**Figure 1:**
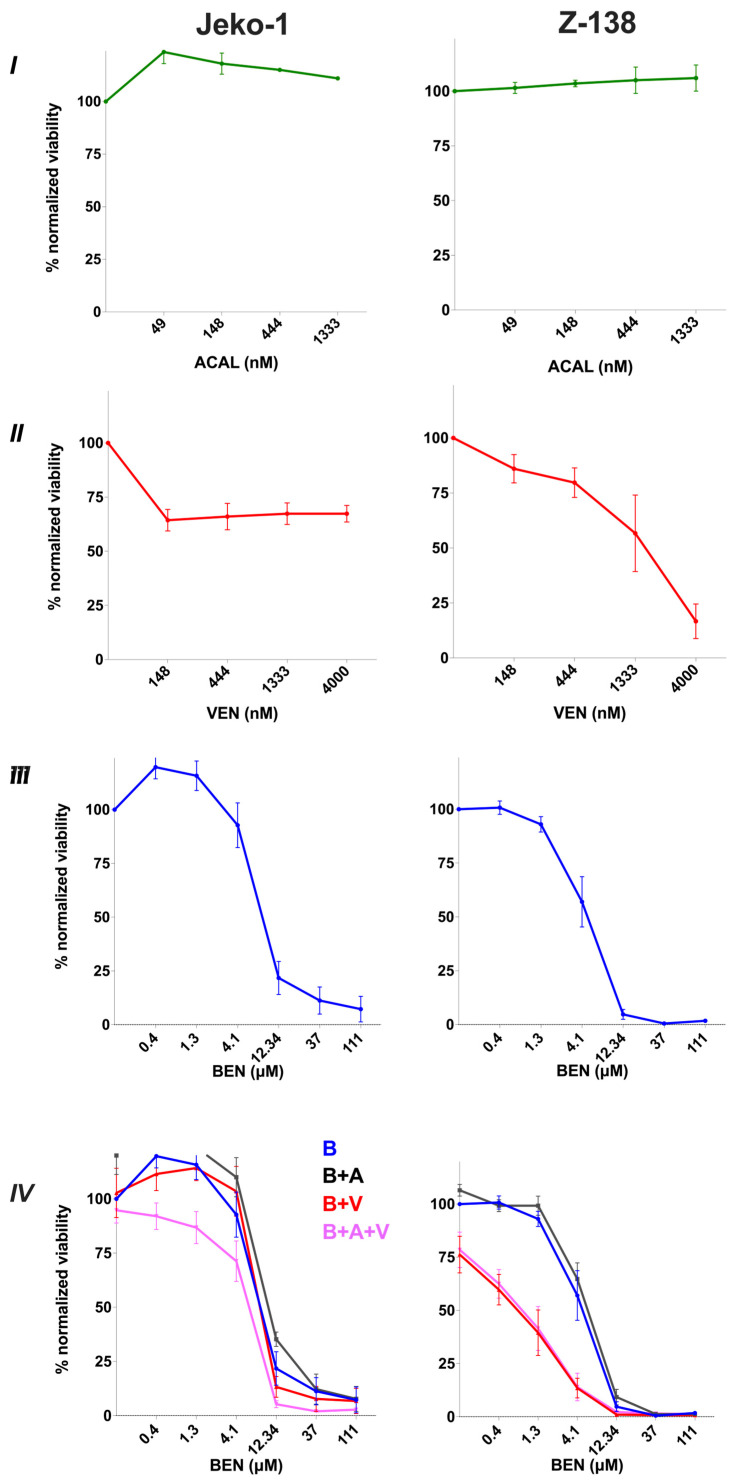
(**I**) Single-agent dose curves of Acalabrutinib (A), (**II**) Venetoclax (V) and (**III**) Bendamustine (B) on the human MCL cell lines Jeko-1 and Z-138. Here, 30,000 cells were treated with increasing concentrations of A, V or B alone for 72 h. (**IV**) Combination dose curves of A and/or V with B. Jeko-1 and Z-138 cells were incubated with increasing concentrations of B alone or in combination with a constant concentration of 1 μM of A, V or both A + V for 72 h. Normalized % viability was measured with the CellTiter 96 AQueous One Solution Cell Proliferation (MTS) assay. Data represent mean +/− S.E.M. from 4 independent experiments. nM = nanomolar; μM = micromolar.

We first performed dose-escalation studies to evaluate the effects of each agent individually against the Jeko-1 and Z-138 cell lines. Both cell lines were found to be unresponsive to ACAL monotherapy even at concentrations approaching the clinical Cmax [28] (Figure 1I). Jeko-1 cells exhibited a sustained resistance to Ven (IC50 not reached) whereas Z-138 showed a response to VEN at higher concentrations (IC50 = 1745 nM, [95% CI: 923–2479 nM]) (Figure 1II). In contrast, BEN significantly reduced the viability of both Jeko-1 and Z-138, with IC50 values of 6.5 μM (95% CI: 5.1–9.4 μM) and 4.5 μM (95% CI: 3.8–5.3 μM), respectively, both responding to low, clinically achievable concentrations (Figure 1III) [29]. Therefore, we sought to determine whether combining VEN with BEN in the presence or the absence of ACAL could further reduce cell proliferation in vitro. For these combination assays we used a constant concentration of 1 μM of ACAL and/or VEN, concentrations that are high but clinically achievable alongside increasing concentrations of BEN [28,30,31,32]. We found that adding constant ACAL to BEN did not improve efficacy in either cell line (BEN IC50 = 8.2 μM [95% CI: not determined] in Jeko-1 and 5 μM [95% CI: 4.3–5.8 μM] in Z-138 respectively (Figure 1IV). Interestingly, although the addition of constant VEN had no impact against Jeko-1 (IC50 = 6.7 μM [95% CI: not determined]), it significantly enhanced the sensitivity of Z-138 to BEN (BEN IC50 = 1.4 μM [95% CI: 0.8–2 μM]) (Figure 1IV). Similarly, combining BEN with both constant ACAL and VEN only marginally reduced the BEN IC50 in Jeko-1 (5.6 μM [95% CI: not determined]) but had a notable effect in Z-138 (BEN IC50 = 1.7 μM [95% CI: 0.7–2.3 μM]) (Figure 1IV). These results suggest that the combination of VEN and ACAL can enhance the efficacy of BEN particularly in Z-138 cells, with VEN contributing most to the observed additional effect.

### 3.2. Bendamustine Combined with Venetoclax +/− Acalabrutinib Increases Apoptosis and Cell Death

**Figure 2:**
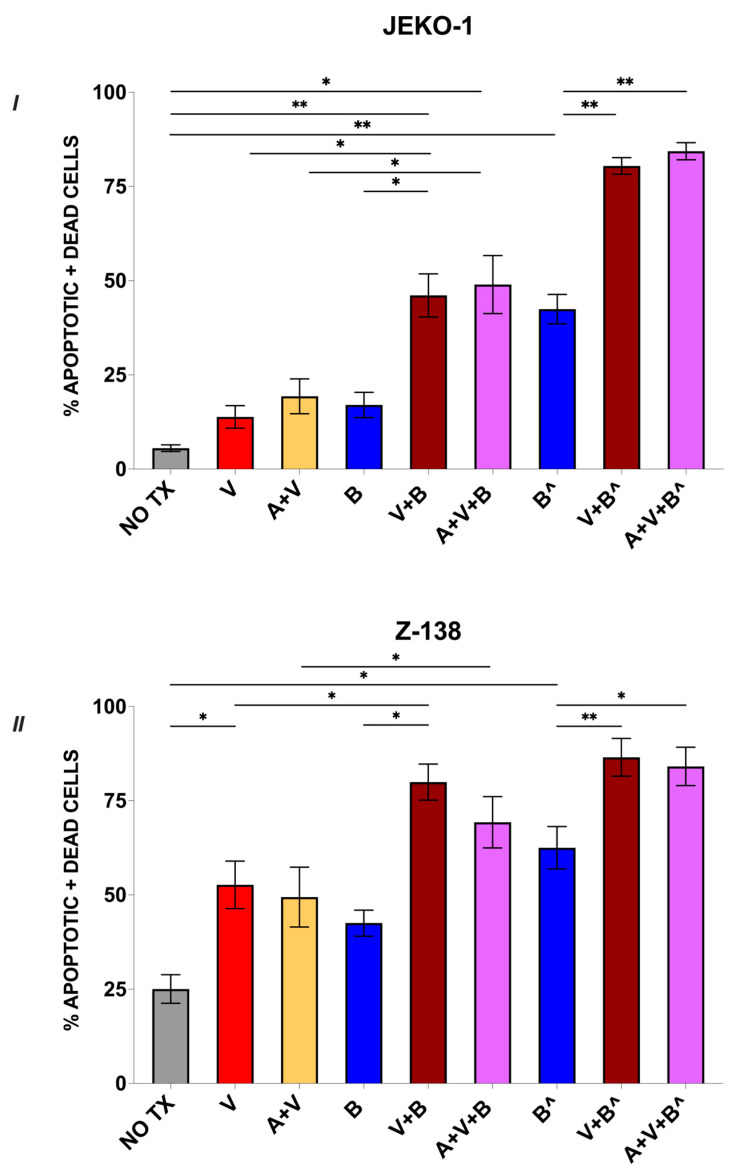
Bendamustine (BEN-B) combined with Venetoclax (VEN-V) +/− Acalabrutinib (ACAL-A) increases apoptosis and cell death. (**I**) Jeko-1 was treated for 24 h with different doses of BEN (B = 12.3 μM and B^ = 37 μM) alone or in combination with 1 μM of VEN, with or without ACAL 1 μM. (**II**) Z-138 cells were treated for 24 h with different doses of BEN (B = 1.3 μM or B^ = 4.1 μM) alone or in combination with 1 μM of VEN, with or without ACAL 1 μM. The percentage of dead and apoptotic cells was assessed with the use of Annexin V/Propidium Iodide staining. Data represent the mean +/− S.E.M. from 5 independent experiments. * *p* < 0.05, ** <0.01, μM = micromolar.

To explore the mechanisms underlying the differential growth inhibition observed across treatment conditions, we performed flow cytometry analysis using Annexin V/PI staining 24 h after treatment. Specifically, we assessed whether the addition of VEN with or without ACAL to increasing doses of BEN could enhance apoptosis and overall cell death. In Jeko-1 cells, we found that VEN at 1 μM, with or without 1 μM of ACAL, led to non-significant enhancements in cell death (13.8% and 19.3% respectively). Similarly, treatment with BEN alone at a concentration of 12.34 μM resulted in a non-significant increase in the percentage of apoptotic and dead cells compared to the untreated condition (17%) (Figure 2I). However, combining this concentration of BEN (12.34 μM) with VEN or with ACAL plus VEN significantly increased cell death (46% and 48.9%, respectively) compared to the untreated condition (*p* = 0.009 and *p* = 0.02 respectively), with BEN and VEN also differing compared to BEN alone (*p* = 0.03). Moreover, the BEN and VEN combination outperformed the VEN monotherapy (*p* = 0.01) with a similar effect being noted when comparing the combination of BEN with ACAL and VEN to the ACAL plus VEN condition (*p* = 0.01) (Figure 2I). We further evaluated cytotoxicity at a higher supraphysiologic concentration of BEN (37 μM). At this dose, BEN alone significantly increased cell death compared to the untreated control (42.4%, *p* = 0.003) as well as the VEN monotherapy (*p* = 0.03), but not compared to the ACAL plus VEN condition (*p* = 0.1). Notably, the addition of VEN with or without ACAL further potentiated BEN-induced cytotoxicity (80.4% and 84.3%), with significant differences compared to BEN monotherapy, (*p* = 0.007 and *p* = 0.003, respectively) (Figure 2I).

Given the higher sensitivity of Z-138 to BEN observed in the MTS assays, we performed the Annexin V/PI cytotoxicity assays using lower BEN concentrations (1.3 and 4.1 μM) while maintaining the same concentrations of VEN and ACAL (both at 1 μM) (Figure 2II). In the absence of BEN, VEN significantly increased the apoptotic rate compared to the untreated condition (52.6%, *p* = 0.03) with a comparable, though non-significant, cytotoxicity seen when VEN was combined with ACAL (49.4%, *p* = 0.1). As expected from the MTS results, BEN monotherapy at a low concentration of 4.1 μM significantly increased cell death compared to the untreated control (62.5%, *p* = 0.03). Notably, combining this dose of BEN with VEN further enhanced apoptosis and cell death (86.5%) to a level significantly exceeding both BEN monotherapy (*p* = 0.005) and VEN monotherapy (*p* = 0.04). The triple combination of BEN, VEN and ACAL also significantly outperformed BEN alone (84.1%, *p* = 0.02), but not the ACAL plus VEN condition (*p* = 0.09) (Figure 2II). When the BEN concentration was reduced three-fold to 1.3 μM, it still increased cytotoxicity (42.5%) but lacked statistical significance compared to untreated cells (*p* = 0.06). The triple combination (BEN + ACAL + VEN) significantly enhanced cytotoxicity compared to the combination of ACAL with VEN (69.2%, *p* = 0.02) and the untreated condition (*p* = 0.01), but not compared to BEN alone (*p* = 0.1). Interestingly, the BEN plus VEN combination still resulted in significantly greater cytotoxicity (79.9%) compared to BEN alone (*p* = 0.02) and the VEN monotherapy (*p* = 0.02) (Figure 2II). Overall, these results suggest that BEN effectively induces apoptosis in both cell lines, and that its apoptotic effects are maintained even at lower doses, when combined with VEN with or without ACAL.

### 3.3. Oral Bendamustine Significantly Improves Survival in MCL-Bearing Mice Alone or in Combination with Venetoclax

**Figure 3:**
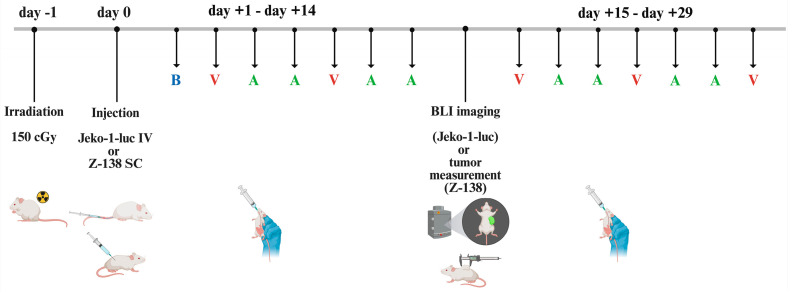
In vivo experimental layout for Jeko-1-Luc intravenous model and for Z-138 subcutaneous model. B = Bendamustine, A = Acalabrutinib, V = Venetoclax, SC = subcutaneous, IV = intravenous, BLI = bioluminescence imaging. Created in https://Biorender.com.

Our group previously demonstrated that the low dose oral BEN (17.65 mg/kg) achieves a Cmax of 14 μM, which is a clinically achievable concentration [27,33]. Based on this, we conducted in vivo experiments using NSG mice bearing either intravenously inoculated Jeko-1-Luc cells or subcutaneously implanted Z-138 cells to assess the therapeutic effects of oral BEN, both as monotherapy and in combination with VEN ± ACAL (Figure 3). The selection of treatment regimens for in vivo evaluation was guided by in vitro synergy analyses based on the IC50 shifts. Due to the pronounced cytotoxicity seen with both BEN + VEN as well as the triple combination of BEN with ACAL and VEN Z-138 in vitro, we assessed both regimens in vivo against this cell line. In contrast, since only the triple combination regimen demonstrated a higher trend towards reducing the IC50 of Bendamustine against Jeko-1, it was the only combination regimen that we tested in the Jeko-1 xenograft model.

**Figure 4:**
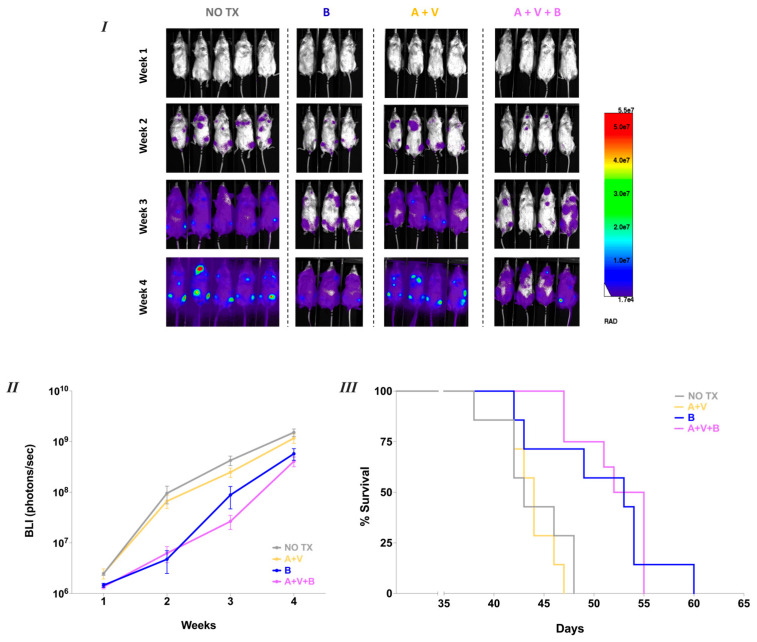
Oral B = Bendamustine inhibits tumor progression in NSG mice engrafted with Jeko-1-Luc. (**I**) BLI images from one representative experiment using Jeko-1-Luc at the indicated time points. A = Acalabrutinib, V = Venetoclax. (**II**) BLI data monitored once weekly following Jeko-1-luc inoculation. Data were analyzed using a mixed linear model with Tukey’s post-hoc test to assess the differences between the groups. *p* values: NO TX vs. AV = ns, B < 0.02, A + V + B < 0.02; A + V vs. B < 0.02, A + V + B < 0.02. (**III**) Kaplan–Meier survival curve of mice inoculated with Jeko-1-Luc that received vehicle controls (NO TX) (*n* = 7), A + V (*n* = 7), B (*n* = 7), A + V + B (*n* = 8). Data were pooled from 2 independent experiments. Comparison between groups was done using the log-rank test. *p* values: NO TX vs. A + V = ns, B < 0.02, A + V + B < 0.002; A + V vs. B < 0.02, A + V + B < 0.002; B vs. A + V + B = ns.

In the Jeko-1 xenograft model, tumor burden was monitored by measuring the bioluminescence imaging (BLI) signal (Figure 4I). No significant differences in tumor burden or median survival were observed between the ACAL + VEN group (median survival: 44 days) and the untreated group (43 days) (Figure 4II,III). In contrast, starting three weeks after tumor inoculation, mice treated with oral BEN exhibited significantly lower BLI signals compared to both to the untreated cohort (*p* = 0.01) and the ACAL + VEN group (*p* = 0.007). Similar reductions in BLI signal were also observed in the group receiving the combination of BEN with ACAL and VEN (*p* = 0.01 and *p* = 0.02, respectively). Regarding survival, oral BEN monotherapy conferred a significant advantage over both the untreated group and the ACAL + VEN group (median survival 53 days, *p* = 0.01 and *p* = 0.01 for both comparisons) (Figure 4III). However, combining BEN with ACAL + VEN did not further extend survival beyond that achieved with BEN monotherapy (median survival 53.5 days).

**Figure 5:**
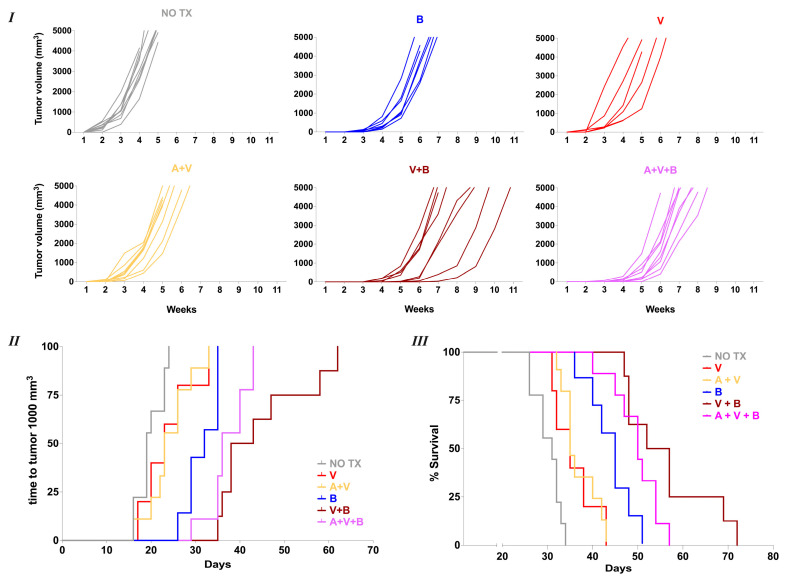
Combining Venetoclax (V) with oral Bendamustine (B), with or without Acalabrutinib (A), significantly suppresses Z-138 tumor progression in NSG mice. (**I**) Individual tumor measurements of Z-138 xenograft mice receiving vehicle control (NO TX) (*n* = 9), A + V (*n* = 9), V (*n* = 5), B (*n* = 7), A + V + B (*n* = 9) or V + B (*n* = 8). Mice were assessed twice weekly for tumor growth. Each time point represents the average of two weekly measurements. (**II**) Kaplan–Meier analysis of time to a tumor size of 1000 mm^3^. *p* values: NO TX vs. V = ns, A + V < 0.03, B, V + B, A + V + B < 0.0001; V vs. A + V = ns, B < 0.03, V + B < 0.0001, A + V + B < 0.002; A + V vs. B < 0.009, V + B < 0.0001, A + V + B < 0.001; B vs. V + B < 0.0005, A + V + B < 0.005; V + B vs. A + V + B = ns. (**III**) Survival of mice with subcutaneous Z-138 xenografts. Mice were euthanized when the tumor volume reached or exceeded 5000 mm^3^. *p* values: NO TX vs. V < 0.03, A + V < 0.0002, B, V + B, A + V + B < 0.0001; V vs. A + V = ns, B < 0.02, V + B < 0.0001, A + V + B < 0.0002; A + V vs. B < 0.007, V + B < 0.0001, A + V + B < 0.0001; B vs. V + B < 0.003, A + V + B < 0.04; V + B vs. A + V + B = ns. Data were pooled from 3 independent experiments. Comparisons between groups were performed using the log-rank test.

In the Z-138 xenograft model, tumor volume was monitored to assess whether different treatment regimens could significantly delay tumor establishment and/or growth, ultimately impacting overall survival. Mice in the untreated group reached a tumor volume of 1000 mm^3^ volume by day +19 and had a median survival of 31 days (Figure 5I–III). Treatment with VEN combined with ACAL delayed tumor progression to 1000 mm^3^ (23 days, *p* = 0.02) and modestly improved overall survival (median survival 35 days, *p* = 0.0002). A similar increase in survival was observed with VEN monotherapy (median survival 35 days, *p* = 0.02). BEN monotherapy significantly suppressed tumor growth, delaying the time to reach 1000 mm^3^ to 32 days. This was not only significant compared to untreated mice (*p* < 0.0001) but was superior to both the VEN + ACAL group (*p* = 0.008) and the VEN monotherapy group (*p* = 0.02), resulting in a markedly improved median survival of 45 days (*p* = 0.01 and *p* = 0.006, respectively). Combining BEN with VEN, either with or without of ACAL, produced even more pronounced effects. Tumors reached 1000 mm^3^ at day 40.5 (*p* = 0.0005) and day 36 (*p* = 0.004) in the BEN + VEN and BEN + VEN + ACAL groups, respectively, with corresponding median survivals of 54.5 days (*p* = 0.002) and 50 days (*p* = 0.03). These results demonstrate that while VEN with or without ACAL provides modest tumor control in vivo against MCL, oral BEN exhibits profound antitumor efficacy both as a monotherapy and in combination regimens. Furthermore, the coadministration of BEN with VEN, with or without ACAL, is feasible and can significantly enhance antitumor effects, depending on the specific characteristics of the MCL cell line.

## 4. Discussion

Our group recently demonstrated that the oral formulation of BEN exhibits good bioavailability and comparable efficacy to the intravenous form, highlighting its potential for broader application in hematological malignancies [27]. In this study we evaluated the feasibility of oral BEN as a single agent and in combination with other oral therapies in the setting of MCL. We found that oral BEN is effective even as a monotherapy and could be combined with other oral agents. Notably, the combination of oral BEN and VEN resulted in a cell-line-dependent synergistic effect.

MCL is an aggressive B-cell malignancy that remains incurable, with nearly all patients eventually relapsing [3,25]. Recent therapeutic advances in R/R MCL have centered on Bruton tyrosine kinase (BTK) inhibitors, which have demonstrated efficacy across several clinical trials [10,11]. ACAL, a second-generation irreversible BTKi, is FDA-approved for R/R MCL and has shown improved overall response rates (ORR) and complete response rates (CRR) compared to the first-generation BTKi ibrutinib [11,34]. However, resistance to BTKi therapy remains a significant clinical issue, occurring in up to one-third of patients even as primary resistance [35,36]. Risk factors for BTK resistance include TP53 mutations and multiple prior therapies [37]. Clinical studies have shown that R/R MCL patients with TP53 mutations or blastoid histology are more likely to develop resistance to BTK inhibition and have poorer outcomes [10]. In our experiments, both Jeko-1 and Z-138 cell lines were resistant to ACAL monotherapy even at high doses. Previous literature has also shown that Z-138 did not respond to ACAL, while Jeko-1 cells have limited sensitivity [38,39]. This resistance is likely attributable to their underlying biology: Z-138 originates from a patient with blastoid variant MCL, and Jeko-1 lacks functional p53 [40,41,42].

VEN, a selective oral BCL-2 inhibitor, has shown encouraging results in clinical trials for R/R MCL [43]. As a monotherapy or in combination, VEN achieved an ORR of 40% and a median progression-free survival (PFS) of 3.7 months in high-risk MCL patients [44]. However, several MCL cell lines, including Jeko-1 and Z-138, exhibit intrinsic resistance to VEN due to the high expression of other anti-apoptotic proteins, such as B-cell lymphoma-extra-large (Bcl-xL) [42,45,46]. A recent study indicated that Bcl-xL is associated with VEN resistance, which could be overcome by concurrent Bcl-2 and BCL-xL blockade [37,47]. Interestingly, Z-138 has shown greater sensitivity to BCL-2 inhibition with VEN and to combined BCL-2 and protein arginine methyltransferase 5 (PRMT5) inhibition [48]. Consistent with prior studies, our in vitro data show that both Jeko-1 and Z-138 were resistant to VEN, with Z-138 demonstrating partial sensitivity only at high doses. Additionally, apart from Bcl-xL upregulation, TP 53 mutation might also be an underlying mechanism behind the limited response of Jeko-1 cells to VEN [42,49]. This is supported by a phase 1 study in patients with R/R MCL, which noted that TP53 alterations were observed significantly more frequently in patients who progressed despite therapy with VEN [50].

Consequently, we evaluated the in vitro effects of BEN as a single agent against Jeko-1 and Z-138, two MCL cell lines that model the relapsed/refractory disease state. BEN treatment resulted in a dose-dependent reduction in cell proliferation and an increase in apoptotic and dead cells in both lines, with Z-138 exhibiting greater sensitivity than Jeko-1. We then conducted combination assays using BEN in conjunction with high, fixed concentrations of Venetoclax (VEN) and/or Acalabrutinib (ACAL) to determine whether these agents produce additive or synergistic effects. Using Annexin V/PI flow cytometry assays, we found that the combination of VEN + BEN with or without ACAL induced significant cytotoxicity in Jeko-1 cells at 24 h, particularly when BEN was used at higher concentrations approximating clinically reported Cmax [29] and similar to levels we previously observed following a single oral dose of 17.65 mg/kg [27]. However, by 72 h, the cytotoxic effects in Jeko-1 were not sustained, and the IC50 of BEN in the triple combination (ACAL + VEN + BEN) was only marginally and non-significantly lower than with BEN monotherapy. In contrast, in Z-138 cells, the combination of VEN and BEN, with or without ACAL, resulted in a marked reduction in metabolic activity at 72 h and significantly enhanced BEN efficacy. This was evidenced by lower BEN IC50 values in the VEN + BEN and ACAL + VEN + BEN groups compared to BEN alone or ACAL + BEN, with non-overlapping 95% confidence intervals. These findings have been corroborated by 24 h Annexin V/PI flow cytometry, where the VEN + BEN combination was highly effective against Z-138, outperforming both the VEN and BEN monotherapies. Notably, the addition of ACAL to BEN (in the absence of VEN) did not improve cytotoxicity in either cell line. Previous studies have shown that BEN combined with IBR synergized Jeko-1 by promoting apoptosis [51], and that IBR + BEN + rituximab (RTX) improved progression-free survival (PFS) in previously untreated MCL patients [52]. Furthermore, Philips et al. reported that adding ACAL to the BEN-RTX regimen was both safe and effective, particularly in treatment-naïve MCL [53]. However, our findings suggest that, in the relapsed/refractory setting, and possibly due to the intrinsic biology of the cell lines, combining a BTK inhibitor with BEN may not necessarily yield additional cytotoxic benefit.

Oral chemotherapy offers convenience and flexibility for many cancer patients, minimizing disruptions to daily life by reducing the need for frequent hospital visits [54]. This is especially important for the elderly population, which constitutes the majority of MCL patients [55,56]. However, non-adherence, whether due to side effects, forgetfulness, or pharmacokinetic drug interactions, can significantly impact oral anticancer therapies [57,58]. To mitigate potential toxicities in our murine in vivo experiments, we restricted oral gavage treatments to one agent per day, aiming to reduce the risk of unforeseen adverse effects. Given the high incidence of toxicity reported in clinical trials combining BEN + VEN + IBR, we reduced the BEN dose to 30 mg/kg in our in vivo models [24]. We then conducted experiments using NSG mice intravenously injected with Jeko-1-Luc cells or subcutaneously injected with Z-138 cells. The treatment regimens included oral BEN monotherapy, or combination therapy with VEN, with or without ACAL. The selection of combination treatments for in vivo validation was guided by the IC-50 shifts, derived from our in vitro MTS assays, to prioritize combinations with the highest potential synergy. In the Jeko-1-Luc intravenous model, we tested the triple combination ACAL + VEN + BEN and compared it to BEN monotherapy and the ACAL + VEN control group. Interestingly, oral BEN significantly reduced tumor burden and prolonged tumor-free survival in vivo. Consistent with our in vitro findings from both Annexin-PI and MTS assays, the ACAL + VEN combination was ineffective, resulting in survival comparable to that of untreated controls. Furthermore, the triple combination (ACAL + VEN + BEN) did not outperform BEN monotherapy, mirroring the small, non-significant reduction in BEN IC50 observed in vitro, and led to similar tumor-free survival. These results underscore that oral BEN is effective as a single agent against Jeko-1-Luc, even in the absence of combination partners. Although BEN is traditionally combined with RTX, prior studies have demonstrated that BEN monotherapy, administered every four weeks, retains efficacy in R/R MCL patients [59]. A multicenter Japanese study further reported a 100% overall response rate (ORR) with BEN monotherapy in R/R MCL, emphasizing the importance of treatment continuation and supporting the feasibility of dose reductions to maintain efficacy [60]. In our Z-138 subcutaneous tumor model, we expanded the treatment groups to include VEN alone, VEN + BEN, and their combinations with ACAL. In line with the greater in vitro sensitivity of Z-138 to VEN, mice treated with VEN ± ACAL survived longer than untreated controls. Similarly to the Jeko-1-Luc IV model, BEN monotherapy was highly effective, inducing profound tumor growth suppression and extending survival. A previous study demonstrated that VEN combined with BEN + RTX delayed tumor progression in mice bearing Granta-519, another MCL cell line. Notably, 50% of mice receiving the VEN + BEN + RTX combination achieved complete responses, a result not observed with BEN + RTX alone. The authors suggested that the addition of VEN may enhance the depth of response, potentially allowing for intermittent VEN administration [61]. In our experiments, VEN was administered only two to three times per week, yet in the Z-138 model, the combination of VEN with BEN significantly improved tumor growth delay and survival compared to BEN alone, regardless of the presence of ACAL. This supports our in vitro evidence of synergy between BEN and VEN, and highlights the therapeutic promise of this all-oral regimen against aggressive MCL.

Our study has several limitations. First, we restricted treatment to a single oral medication per day, which does not directly reflect clinical practice or trial protocols, where multiple agents are often administered concurrently. Additionally, to maintain the focus on oral therapies, we excluded rituximab, which is typically administered intravenously and is commonly used in combination with BEN in clinical settings. Furthermore, our experiments were limited to two MCL cell lines—Jeko-1 and Z-138—that represent aggressive and relapsed/refractory subtypes of MCL. As such, the findings may not fully capture the spectrum of MCL biology. It remains to be determined whether the combination of oral BEN with other agents is similarly effective against treatment-naïve disease or other molecular subtypes of R/R MCL, particularly in patient-derived samples or primary xenograft models.

Overall, our data suggest that the combination of oral BEN and VEN, with or without ACAL, is a potent therapeutic strategy in MCL. However, the extent of synergy may be cell line- or subtype-dependent, underscoring the need for further investigation to define the contexts in which this combination is most effective.

## 5. Conclusions

In summary, our results demonstrate that the oral formulation of BEN exhibits strong antitumor activity in MCL models both as a monotherapy and in combination with VEN and/or ACAL. Given that BEN remains a standard-of-care therapy for MCL, these findings provide a rationale for the clinical exploration of this oral formulation, either alone or in combination with targeted agents currently in use or under investigation for the treatment of MCL.

## Data Availability

The data that support the findings of this study are available from the corresponding author, E.K., upon reasonable request.

## Abbreviations

The following abbreviations are used in this manuscript:

BEN	Bendamustine
ACAL	Acalabrutinib
VEN	Venetoclax
MCL	Mantle-cell lymphoma
R/R	Relapsed/Refractory
BTKi	Bruton’s tyrosine kinase inhibitor
RTX	Rituximab
Bcl-2	B-cell lymphoma 2
DMSO	Dimethylsulfoxide
IC50	Half max inhibitory concentration
PI	Propidium iodide
Bcl-xL	B-cell lymphoma-extra large
MCL-1	Myeloid cell leukemia 1
Cmax	Maximum plasma concentration
IBR	Ibrutinib
OS	Overall survival
ORR	Overall response rate
PFS	Progression-free survival
CR	Complete Response
SEM	Standard error of the means
PBS	Phosphate buffer saline
ANOVA	Analysis of variance
BLI	Bioluminescence imaging
μM	Micromolar
nM	Nanomolar
IV	Intravenous
SC	Subcutaneous
PO	Per os
Cgy	Centigray
CIT	Chemoimmunotherapy
CI	Confidence interval
HEPES	4-(2-hydroxyethyl)-1-piperazineethanesulfonic acid
RPMI 1640	Roswell Park Memorial Institute
PEG	Polyethylene glycol
NSG mice	(NOD.Cg-Prkdcscid Il2rgtm1Wjl/SzJ) mice
Phosal50-PG	Phosphatidylcholine in propylene glycol, content ≥ 50.0%
